# *O*,*O*′-Diisopropyl *S*-[2-(benzene­sulfon­amido)­eth­yl]phospho­rodithio­ate

**DOI:** 10.1107/S160053681105536X

**Published:** 2012-01-07

**Authors:** Hai-Feng Wu, Xin-Yi Liu, Fan-Hua Zhang, Yun-Xiao He

**Affiliations:** a Sinochem Ningbo (Group) Co. Ltd, Ningbo, Zhejiang 315000, People’s Republic of China

## Abstract

The mol­ecular conformation of the title compound, C_14_H_24_NO_4_PS_3_, the selective herbicide bensulide, is stabilized by a weak intra­molecular C—H⋯S inter­action. In the crystal, chains are formed through inter­molecular N—H⋯S hydrogen bonds.

## Related literature

For applications of *N*-(β-diorganodithio­phospho­ryleth­yl) aryl and alkyl sulfonamides in the field of agrochemicals, see: Llewellyn & Chester (1963 ▶). Bensulide is a selective organophosphate herbicide which is mainly used on vegetable crops such as carrots, cucumbers, peppers and melons, see: Meister (1992 ▶). For the synthesis, see: Llewellyn & Jeffrey (1978 ▶).
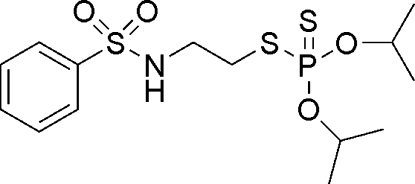


## Experimental

### 

#### Crystal data

C_14_H_24_NO_4_PS_3_
*M* *_r_* = 397.49
Monoclinic, 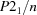

*a* = 8.6431 (17) Å
*b* = 24.465 (5) Å
*c* = 9.875 (2) Å
β = 104.99 (3)°
*V* = 2017.1 (8) Å^3^
*Z* = 4
Mo *K*α radiation
μ = 0.46 mm^−1^
*T* = 293 K
0.37 × 0.35 × 0.27 mm

#### Data collection

Rigaku R-AXIS RAPID CCD diffractometer
Absorption correction: multi-scan (*ABSCOR*; Higashi, 1995 ▶) *T*_min_ = 0.843, *T*_max_ = 0.883
19632 measured reflections
4605 independent reflections
3083 reflections with *I* > 2σ(*I*)
*R*_int_ = 0.029

#### Refinement

*R*[*F* ^2^ > 2σ(*F* ^2^)] = 0.041
*wR*(*F* ^2^) = 0.127
*S* = 1.11
4605 reflections
208 parameters
H-atom parameters constrained
Δρ_max_ = 0.35 e Å^−3^
Δρ_min_ = −0.40 e Å^−3^

### 

Data collection: *RAPID-AUTO* (Rigaku, 1998 ▶); cell refinement: *RAPID-AUTO*; data reduction: *CrystalStructure* (Rigaku/MSC, 2004 ▶); program(s) used to solve structure: *SHELXS97* (Sheldrick, 2008 ▶); program(s) used to refine structure: *SHELXL97* (Sheldrick, 2008 ▶); molecular graphics: *ORTEPII* (Johnson, 1976 ▶); software used to prepare material for publication: *SHELXL97*.

## Supplementary Material

Crystal structure: contains datablock(s) global, I. DOI: 10.1107/S160053681105536X/zs2173sup1.cif

Structure factors: contains datablock(s) I. DOI: 10.1107/S160053681105536X/zs2173Isup2.hkl

Supplementary material file. DOI: 10.1107/S160053681105536X/zs2173Isup3.cml

Additional supplementary materials:  crystallographic information; 3D view; checkCIF report

## Figures and Tables

**Table 1:** Hydrogen-bond geometry (Å, °)

*D*—H⋯*A*	*D*—H	H⋯*A*	*D*⋯*A*	*D*—H⋯*A*
N—H0*A*⋯S1^i^	0.86	2.86	3.496 (2)	132
C7—H7*B*⋯S1	0.97	2.83	3.447 (3)	122

Symmetry code: (i) 
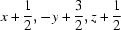
.

**Fig. 1.**
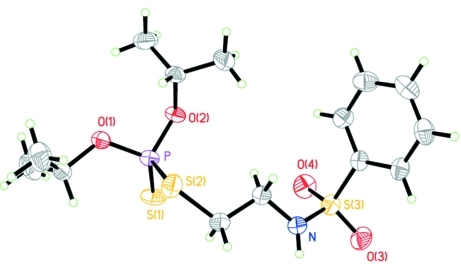
An ORTEP view of the title compound with displacement ellipsoids drawn at the 35% probability level.

**Fig. 2.**
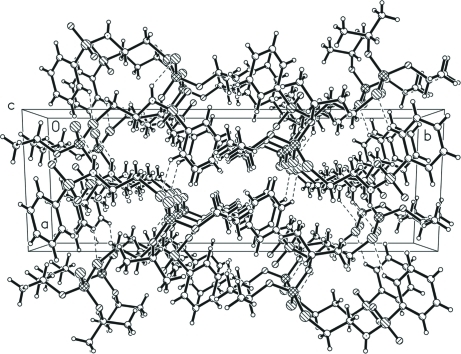
Part of the crystal packing of (I). Weak intermolecular interactions are shown as dashed lines.

**Fig. 3.**
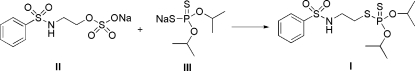
Reaction scheme for the synthesis of (I).

## supplementary crystallographic information

### Comment

*N*-(β-Diorganodithiophosphorylethyl) aryl and alkyl sulfonamides are known for their applications in the field of agrochemicals because of their significant biological properties (Llewellyn *et al.*, 1963). The title compound C_14_H_24_NO_4_PS_3_ (I), with the common name bensulide, is a selective organophosphate herbicide which is mainly used on vegetable crops such as carrots, cucumbers, peppers and melons (Meister, 1992). This typical organic phosphorus compound is one of our plant products that can be synthesized by combining the sodium salt of 2-(phenylsulfonamido)ethyl sulfate (II) and *O,O'*-diisopropyl phosphorodithioate (III) (Llewellyn *et al.*, 1978) (Fig. 3).

In the title compound (Fig. 1), bond distances and angles are as expected. The P atom is coordinated by two S atoms and two O atoms. The O1—P—S1, S1—P—S2 and O2—P—S1 bond angles [117.91 (7), 114.48 (5), 111.46 (7)°, respectively] are larger than those for angles O2—P—S2, O1—P—O2 and O1—P—S2 [108.85 (8), 102.35 (9), 100.58 (7)°, respectively], indicating a distorted tetrahedral configuration. The molecular conformation is stabilized by weak intramolecular C—H···S and C—H···O interactions and one-dimensional chains are formed through intermolecular N—H···S hydrogen bonds (Table 1, Fig. 2).

### Experimental

A 30% aqueous solution of sodium 2-(phenylsulfonamido)ethyl sulfate [(II), 0.5mol] was added to a 30% aqueous solution of sodium *O,O'*-diisopropyl phosphorodithioate [(III), 0.5 mol]. Addition of 50% aqueous sodium hydroxide brought the pH to 10.5. The mixture was then heated to 85 °C for 4 h with vigorous stirring after which the reaction flask was cooled to 25 °C. The pH was lowered from 12 to approximately 8 by the addition of concentrated sulfuric acid. The product was extracted with toluene (400 ml), washed with 2% sodium bicarbonate solution followed by a saturated sodium chloride solution, then dried and evaporated. Recrystallization from toluene gave colourless blocks of (I).

### Refinement

Hydrogen atoms were placed in geometrically calculated positions with C—H = 0.93–0.97 Å and were treated using a riding model approximation with *U*_iso_(H) = 1.2*U*_eq_(aromatic C or N) or 1.5*U*_eq_(methyl C).

### Crystal data

**Table:**

C_14_H_24_NO_4_PS_3_	*F*(000) = 840
*M**_r_* = 397.49	*D*_x_ = 1.309 Mg m^−^^3^
Monoclinic, *P*2_1_/*n*	Mo *K*α radiation, λ = 0.71073 Å
Hall symbol: -P 2yn	Cell parameters from 19632 reflections
*a* = 8.6431 (17) Å	θ = 3.3–27.5°
*b* = 24.465 (5) Å	µ = 0.46 mm^−^^1^
*c* = 9.875 (2) Å	*T* = 293 K
β = 104.99 (3)°	Block, colorless
*V* = 2017.1 (8) Å^3^	0.37 × 0.35 × 0.27 mm
*Z* = 4	

### Data collection

**Table:**

Rigaku R-AXIS RAPID CCD diffractometer	4605 independent reflections
Radiation source: fine-focus sealed tube	3083 reflections with *I* > 2σ(*I*)
graphite	*R*_int_ = 0.029
ω scans	θ_max_ = 27.5°, θ_min_ = 3.3°
Absorption correction: multi-scan (*ABSCOR*; Higashi, 1995)	*h* = −11→10
*T*_min_ = 0.843, *T*_max_ = 0.883	*k* = −31→31
19632 measured reflections	*l* = −12→12

### Refinement

**Table:**

Refinement on *F*^2^	Primary atom site location: structure-invariant direct methods
Least-squares matrix: full	Secondary atom site location: difference Fourier map
*R*[*F*^2^ > 2σ(*F*^2^)] = 0.041	Hydrogen site location: inferred from neighbouring sites
*wR*(*F*^2^) = 0.127	H-atom parameters constrained
*S* = 1.11	*w* = 1/[σ^2^(*F*_o_^2^) + (0.0534*P*)^2^ + 0.6749*P*] where *P* = (*F*_o_^2^ + 2*F*_c_^2^)/3
4605 reflections	(Δ/σ)_max_ = 0.001
208 parameters	Δρ_max_ = 0.35 e Å^−^^3^
0 restraints	Δρ_min_ = −0.40 e Å^−^^3^

### Special details

**Table:**

Geometry. All e.s.d.'s (except the e.s.d. in the dihedral angle between two l.s. planes) are estimated using the full covariance matrix. The cell e.s.d.'s are taken into account individually in the estimation of e.s.d.'s in distances, angles and torsion angles; correlations between e.s.d.'s in cell parameters are only used when they are defined by crystal symmetry. An approximate (isotropic) treatment of cell e.s.d.'s is used for estimating e.s.d.'s involving l.s. planes.
Refinement. Refinement of *F*^2^ against ALL reflections. The weighted *R*-factor *wR* and goodness of fit *S* are based on *F*^2^, conventional *R*-factors *R* are based on *F*, with *F* set to zero for negative *F*^2^. The threshold expression of *F*^2^ > σ(*F*^2^) is used only for calculating *R*-factors(gt) *etc*. and is not relevant to the choice of reflections for refinement. *R*-factors based on *F*^2^ are statistically about twice as large as those based on *F*, and *R*- factors based on ALL data will be even larger.

### Fractional atomic coordinates and isotropic or equivalent isotropic displacement parameters (Å^2^)

**Table:**

	*x*	*y*	*z*	*U*_iso_*/*U*_eq_	
P	0.25939 (7)	0.64011 (3)	0.36909 (6)	0.04780 (17)	
S1	0.36714 (8)	0.63940 (4)	0.22205 (8)	0.0740 (2)	
S2	0.40173 (9)	0.66439 (3)	0.56352 (7)	0.0654 (2)	
S3	0.47902 (8)	0.85949 (3)	0.34382 (7)	0.05828 (19)	
N	0.5557 (2)	0.81061 (8)	0.4499 (2)	0.0591 (5)	
H0A	0.6574	0.8085	0.4858	0.071*	
O1	0.19173 (18)	0.58432 (6)	0.40940 (18)	0.0543 (4)	
O2	0.10605 (17)	0.67690 (7)	0.33022 (17)	0.0520 (4)	
O3	0.6077 (2)	0.89486 (8)	0.3366 (2)	0.0812 (6)	
O4	0.3819 (3)	0.83522 (9)	0.2196 (2)	0.0808 (6)	
C1	0.2942 (3)	0.53593 (10)	0.4530 (3)	0.0570 (6)	
H1A	0.4064	0.5460	0.4633	0.068*	
C2	0.2453 (5)	0.49360 (13)	0.3407 (4)	0.0934 (11)	
H2A	0.2640	0.5072	0.2551	0.140*	
H2B	0.1336	0.4855	0.3263	0.140*	
H2C	0.3070	0.4610	0.3686	0.140*	
C3	0.2729 (5)	0.51819 (14)	0.5913 (4)	0.0915 (10)	
H3A	0.3072	0.5469	0.6587	0.137*	
H3B	0.3358	0.4860	0.6219	0.137*	
H3C	0.1620	0.5102	0.5825	0.137*	
C4	−0.0043 (3)	0.68091 (10)	0.4232 (3)	0.0551 (6)	
H4A	0.0481	0.6651	0.5148	0.066*	
C5	−0.1513 (3)	0.64868 (14)	0.3583 (4)	0.0869 (10)	
H5A	−0.1231	0.6110	0.3520	0.130*	
H5B	−0.1995	0.6625	0.2661	0.130*	
H5C	−0.2258	0.6518	0.4150	0.130*	
C6	−0.0328 (4)	0.74050 (12)	0.4411 (4)	0.0874 (10)	
H6A	0.0667	0.7580	0.4862	0.131*	
H6B	−0.1070	0.7449	0.4978	0.131*	
H6C	−0.0763	0.7569	0.3510	0.131*	
C7	0.5288 (3)	0.71656 (11)	0.5167 (3)	0.0647 (7)	
H7A	0.6232	0.7214	0.5941	0.078*	
H7B	0.5640	0.7041	0.4363	0.078*	
C8	0.4462 (3)	0.76989 (10)	0.4830 (3)	0.0601 (6)	
H8A	0.3538	0.7657	0.4035	0.072*	
H8B	0.4088	0.7823	0.5623	0.072*	
C9	0.3490 (3)	0.89472 (9)	0.4243 (2)	0.0505 (5)	
C10	0.4098 (4)	0.93520 (10)	0.5218 (3)	0.0628 (7)	
H10A	0.5180	0.9441	0.5437	0.075*	
C11	0.3071 (4)	0.96202 (12)	0.5857 (3)	0.0766 (8)	
H11A	0.3463	0.9896	0.6502	0.092*	
C12	0.1486 (4)	0.94845 (13)	0.5552 (3)	0.0768 (8)	
H12A	0.0810	0.9666	0.5996	0.092*	
C13	0.0884 (4)	0.90801 (14)	0.4589 (3)	0.0757 (8)	
H13A	−0.0196	0.8988	0.4388	0.091*	
C14	0.1885 (3)	0.88125 (11)	0.3926 (3)	0.0623 (7)	
H14A	0.1481	0.8542	0.3267	0.075*	

### Atomic displacement parameters (Å^2^)

**Table:**

	*U*^11^	*U*^22^	*U*^33^	*U*^12^	*U*^13^	*U*^23^
P	0.0387 (3)	0.0591 (4)	0.0454 (3)	0.0051 (3)	0.0104 (2)	0.0005 (3)
S1	0.0534 (4)	0.1117 (6)	0.0642 (5)	0.0140 (4)	0.0283 (3)	0.0037 (4)
S2	0.0685 (4)	0.0623 (4)	0.0540 (4)	−0.0086 (3)	−0.0046 (3)	0.0056 (3)
S3	0.0680 (4)	0.0611 (4)	0.0495 (4)	−0.0070 (3)	0.0221 (3)	−0.0053 (3)
N	0.0486 (11)	0.0557 (12)	0.0750 (15)	−0.0015 (10)	0.0195 (10)	−0.0034 (10)
O1	0.0462 (8)	0.0532 (9)	0.0630 (11)	0.0029 (7)	0.0130 (8)	−0.0035 (8)
O2	0.0439 (8)	0.0652 (10)	0.0501 (10)	0.0141 (7)	0.0181 (7)	0.0079 (7)
O3	0.0895 (14)	0.0746 (12)	0.0930 (16)	−0.0182 (11)	0.0482 (12)	0.0031 (11)
O4	0.0968 (15)	0.0956 (15)	0.0493 (11)	−0.0054 (12)	0.0176 (10)	−0.0195 (10)
C1	0.0523 (13)	0.0521 (13)	0.0626 (16)	0.0069 (11)	0.0076 (11)	−0.0040 (11)
C2	0.101 (2)	0.0698 (19)	0.095 (3)	0.0190 (18)	−0.001 (2)	−0.0266 (17)
C3	0.121 (3)	0.078 (2)	0.080 (2)	0.010 (2)	0.035 (2)	0.0165 (17)
C4	0.0505 (13)	0.0622 (14)	0.0597 (15)	0.0066 (12)	0.0268 (11)	−0.0014 (12)
C5	0.0572 (16)	0.097 (2)	0.116 (3)	−0.0085 (16)	0.0391 (18)	−0.025 (2)
C6	0.089 (2)	0.0681 (18)	0.123 (3)	0.0079 (17)	0.061 (2)	−0.0056 (18)
C7	0.0460 (13)	0.0616 (15)	0.0792 (19)	0.0012 (12)	0.0031 (12)	0.0021 (13)
C8	0.0564 (14)	0.0547 (14)	0.0730 (18)	−0.0022 (12)	0.0237 (13)	−0.0054 (12)
C9	0.0616 (14)	0.0488 (12)	0.0403 (12)	−0.0011 (11)	0.0118 (10)	0.0044 (9)
C10	0.0729 (17)	0.0571 (14)	0.0581 (16)	−0.0088 (13)	0.0165 (13)	−0.0068 (12)
C11	0.103 (2)	0.0637 (17)	0.0653 (19)	0.0023 (17)	0.0264 (17)	−0.0104 (14)
C12	0.090 (2)	0.0785 (19)	0.0666 (19)	0.0278 (18)	0.0287 (17)	0.0078 (15)
C13	0.0605 (16)	0.096 (2)	0.069 (2)	0.0095 (16)	0.0140 (14)	0.0131 (17)
C14	0.0616 (15)	0.0693 (16)	0.0513 (15)	−0.0022 (13)	0.0059 (12)	−0.0012 (12)

### Geometric parameters (Å, °)

**Table:**

P—O2	1.5654 (16)	C4—H4A	0.9800
P—O1	1.5761 (18)	C5—H5A	0.9600
P—S1	1.9171 (10)	C5—H5B	0.9600
P—S2	2.0811 (11)	C5—H5C	0.9600
S2—C7	1.820 (3)	C6—H6A	0.9600
S3—O4	1.425 (2)	C6—H6B	0.9600
S3—O3	1.4254 (19)	C6—H6C	0.9600
S3—N	1.615 (2)	C7—C8	1.484 (4)
S3—C9	1.761 (3)	C7—H7A	0.9700
N—C8	1.468 (3)	C7—H7B	0.9700
N—H0A	0.8600	C8—H8A	0.9700
O1—C1	1.474 (3)	C8—H8B	0.9700
O2—C4	1.489 (3)	C9—C14	1.381 (3)
C1—C3	1.489 (4)	C9—C10	1.386 (3)
C1—C2	1.496 (4)	C10—C11	1.381 (4)
C1—H1A	0.9800	C10—H10A	0.9300
C2—H2A	0.9600	C11—C12	1.365 (4)
C2—H2B	0.9600	C11—H11A	0.9300
C2—H2C	0.9600	C12—C13	1.377 (4)
C3—H3A	0.9600	C12—H12A	0.9300
C3—H3B	0.9600	C13—C14	1.378 (4)
C3—H3C	0.9600	C13—H13A	0.9300
C4—C5	1.492 (4)	C14—H14A	0.9300
C4—C6	1.497 (4)		
			
O2—P—O1	102.35 (9)	C4—C5—H5A	109.5
O2—P—S1	111.46 (7)	C4—C5—H5B	109.5
O1—P—S1	117.91 (7)	H5A—C5—H5B	109.5
O2—P—S2	108.85 (8)	C4—C5—H5C	109.5
O1—P—S2	100.58 (7)	H5A—C5—H5C	109.5
S1—P—S2	114.48 (5)	H5B—C5—H5C	109.5
C7—S2—P	102.56 (10)	C4—C6—H6A	109.5
O4—S3—O3	120.22 (14)	C4—C6—H6B	109.5
O4—S3—N	107.55 (13)	H6A—C6—H6B	109.5
O3—S3—N	106.68 (13)	C4—C6—H6C	109.5
O4—S3—C9	106.87 (12)	H6A—C6—H6C	109.5
O3—S3—C9	108.84 (12)	H6B—C6—H6C	109.5
N—S3—C9	105.83 (11)	C8—C7—S2	112.74 (18)
C8—N—S3	117.80 (17)	C8—C7—H7A	109.0
C8—N—H0A	121.1	S2—C7—H7A	109.0
S3—N—H0A	121.1	C8—C7—H7B	109.0
C1—O1—P	122.36 (15)	S2—C7—H7B	109.0
C4—O2—P	121.58 (15)	H7A—C7—H7B	107.8
O1—C1—C3	107.1 (2)	N—C8—C7	110.2 (2)
O1—C1—C2	107.8 (2)	N—C8—H8A	109.6
C3—C1—C2	113.6 (3)	C7—C8—H8A	109.6
O1—C1—H1A	109.4	N—C8—H8B	109.6
C3—C1—H1A	109.4	C7—C8—H8B	109.6
C2—C1—H1A	109.4	H8A—C8—H8B	108.1
C1—C2—H2A	109.5	C14—C9—C10	120.4 (2)
C1—C2—H2B	109.5	C14—C9—S3	120.14 (19)
H2A—C2—H2B	109.5	C10—C9—S3	119.4 (2)
C1—C2—H2C	109.5	C11—C10—C9	118.9 (3)
H2A—C2—H2C	109.5	C11—C10—H10A	120.5
H2B—C2—H2C	109.5	C9—C10—H10A	120.5
C1—C3—H3A	109.5	C12—C11—C10	120.7 (3)
C1—C3—H3B	109.5	C12—C11—H11A	119.7
H3A—C3—H3B	109.5	C10—C11—H11A	119.7
C1—C3—H3C	109.5	C11—C12—C13	120.4 (3)
H3A—C3—H3C	109.5	C11—C12—H12A	119.8
H3B—C3—H3C	109.5	C13—C12—H12A	119.8
O2—C4—C5	108.1 (2)	C12—C13—C14	119.8 (3)
O2—C4—C6	106.8 (2)	C12—C13—H13A	120.1
C5—C4—C6	114.7 (2)	C14—C13—H13A	120.1
O2—C4—H4A	109.0	C13—C14—C9	119.7 (3)
C5—C4—H4A	109.0	C13—C14—H14A	120.1
C6—C4—H4A	109.0	C9—C14—H14A	120.1

### Hydrogen-bond geometry (Å, °)

**Table:**

*D*—H···*A*	*D*—H	H···*A*	*D*···*A*	*D*—H···*A*
N—H0A···S1^i^	0.86	2.86	3.496 (2)	132
C7—H7B···S1	0.97	2.83	3.447 (3)	122
C8—H8A···O4	0.97	2.55	2.980 (3)	107
C14—H14A···O4	0.93	2.55	2.908 (4)	103

Symmetry codes: (i) *x*+1/2, −*y*+3/2, *z*+1/2.
